# A Novel Structurally Stable Multiepitope Protein for Detection of HCV

**DOI:** 10.1155/2016/6592143

**Published:** 2016-01-28

**Authors:** Alexsandro S. Galdino, José C. Santos, Marilen Q. Souza, Yanna K. M. Nóbrega, Mary-Ann E. Xavier, Maria S. S. Felipe, Sonia M. Freitas, Fernando A. G. Torres

**Affiliations:** ^1^Laboratório de Biotecnologia de Microrganismos, Universidade Federal de São João Del-Rei, 35501-296 Divinópolis, MG, Brazil; ^2^Departamento de Biologia Celular, Universidade de Brasília, 70910-900 Brasília, DF, Brazil; ^3^Laboratório de Doenças Imunogenéticas e Crônico-degenerativas, Universidade de Brasília, 70910-900 Brasília, DF, Brazil; ^4^Laboratório de Biofísica, Universidade de Brasília, 70910-900 Brasília, DF, Brazil

## Abstract

Hepatitis C virus (HCV) has emerged as the major pathogen of liver diseases in recent years leading to worldwide blood-transmitted chronic hepatitis, liver cirrhosis, and hepatocellular carcinoma. Accurate diagnosis for differentiation of hepatitis C from other viruses is thus of pivotal importance for proper treatment. In this work we developed a recombinant multiepitope protein (rMEHCV) for hepatitis C diagnostic purposes based on conserved and immunodominant epitopes from* core*, NS3, NS4A, NS4B, and NS5 regions of the virus polyprotein of genotypes 1a, 1b, and 3a, the most prevalent genotypes in South America (especially in Brazil). A synthetic gene was designed to encode eight epitopes* in tandem* separated by a flexible linker and bearing a his-tag at the C-terminal end. The recombinant protein was produced in* Escherichia coli* and purified in a single affinity chromatographic step with >95% purity. Purified rMEHCV was used to perform an ELISA which showed that the recombinant protein was recognized by IgG and IgM from human serum samples. The structural data obtained by circular dichroism (CD) spectroscopy showed that rMEHCV is a highly thermal stable protein at neutral and alkaline conditions. Together, these results show that rMEHCV should be considered an alternative antigen for hepatitis C diagnosis.

## 1. Introduction

Hepatitis C virus (HCV) is an important human pathogen affecting 3% of the human population [1]. Chronic infection is a major cause of liver cirrhosis and hepatocellular carcinoma [2]. Seroprevalence studies suggest that at least 170 million individuals have been infected worldwide [1]. The incidence of new HCV infections has decreased in affluent countries owing to screening of blood products, but an increase of global patients is still expected [2, 3]. The HCV genome is represented by a single-stranded positive RNA molecule which encodes a polyprotein of 3010–3033 amino acid residues [4]. The HCV polyprotein is co- and posttranslationally processed to produce several structural and nonstructural polypeptides [5]. Six genotypes and several HCV subtypes are well characterized, with an overall nucleotide diversity of 31%–33% between genotypes and 20%–25% between subtypes [6]. Genotypes 1, 2, and 3 are widely distributed throughout the world and are responsible for almost all cases in America, Europe, and Japan [7]. In Brazil, approximately 2 million acute cases of hepatitis C have been reported [8] with genotype 1 responsible for 60 to 75% of HCV infections [9–11]. Genotype 3 is the second-most prevalent and genotype 2 represents less than 5% of cases.

Several Enzyme Immune Assay (EIA) based diagnostic kits are available in the market for detection of HCV antibodies in the plasma; these are based on peptide antigens (third generation) or recombinant antigens (fourth generation) from both structural and nonstructural regions of the viral protein. The requirement of multiple peptides and/or multiple recombinant proteins for reliable diagnosis of HCV infection may add to the final cost of these EIA kits. Alternatively, the development of multiepitope proteins is an attractive approach to reduce the complexity and the final costs of such diagnostic kits [12]. In this work we have designed a single recombinant multiepitope protein (rMEHCV) consisting of several immunodominant and conserved specific epitopes from structural and nonstructural proteins derived from genotypes 1, 2, and 3, the most prevalent in South America. The recombinant protein was successfully produced and tested for HCV detection in infected patients in Brazil.

## 2. Material and Methods

### 2.1. Strains and Reagents

*Escherichia coli* BL21 (*λ*DE3) pLysS (F^−^ *omp*T* hsd*S_B_ (r_B_^−^  m_B_^−^)* gal dcm* (DE3) pLysS [Cam^r^]) and expression vector pET21a were purchased from Novagen. NiSepharose*™* 6 Fast Flow resin (GE Healthcare) was used to purify rMEHCV. Restriction enzymes were purchased from New England Biolabs. Illustra*™* Plasmid Prep Mini Spin Kit was purchased from GE Healthcare. Secondary antibody-enzyme conjugates, monoclonal anti-poly histidine-alkaline phosphatase (AP) antibody and monoclonal anti-human IgG-HRPO, and HRPO substrate Sigma Fast*™* OPD (o-phenylenediamine dihydrochloride) Peroxidase Substrate Tablet Set were purchased from Sigma-Aldrich. Infected human sera samples were kindly provided by WAMA Diagnóstica (São Carlos, Brazil) and are listed in Table 1. Other reagents of analytical grade were obtained from standard commercial sources.

### 2.2. Design of the Synthetic Gene, Cloning, and Expression

The overall structure of the synthetic gene encoding rMEHCV was based on the construct previously described elsewhere [12] with the inclusion of immunodominant sequences of genotypes prevalent in South America (1a, 1b, and 3a). The sequences used were obtained from GenBank:* core*(1a),* core*(3a), NS3(1a), NS4A(1a), NS4B(1a), and NS5(1a) (accession # AF009606, M62321, and M67463), NS4(1b) and NS5(1b) (accession # D90208, M58335) and from the website https://euhcvdb.ibcp.fr/euHCVdb/. The length of individual epitopes varied from 16–48 amino acid residues and each one was separated by a flexible linker (Gly-Ser-Gly-Ser-Gly). The synthetic gene was custom synthesized by Epoch Biosciences with codon adaptation for* E. coli* and was cloned as a* Nde*I/*Xho*I fragment into pET21a in-frame with a C-terminal histidine tag in order to allow protein purification by affinity chromatography. The resulting plasmid was used to transform* E. coli* BL21 (DE3) competent cells and selection was performed on LB agar plates containing 100 *μ*g/mL ampicillin. An individual colony was inoculated in 5 mL 4YT (32 g/L Bactotryptone, 20 g/L yeast extract, 5 g/L NaCl, pH 7.2) containing 100 *μ*g/mL ampicillin and allowed to grow overnight at 37°C under agitation (200 rpm). One milliliter of the preculture was transferred to 20 mL 4YT in a 250 mL E-flask. The culture was grown in the same conditions described above until an OD_600_ of 0.6 when 1 mM IPTG was added. The induced culture was harvested by centrifugation at 6000 ×g for 15 min at 4°C and the pellet was stored at −80°C.

### 2.3. Purification of rMEHCV

The frozen pellet was resuspended in 1 mL Lysis Buffer (8 M urea, 50 mM NaH_2_PO_4_, 300 mM NaCl, 10 mM imidazole, pH 8.0) following incubation at 4°C for 16 h. After that, cell suspension was sonicated (5 pulses of 10 seconds with 1-minute intervals) using Vibra Cell sonicator (Sonics & Materials, Inc.) and incubated on ice for 2 h following centrifugation at 6000 ×g for 15 min at 4°C. The supernatant was added to 0.5 mL Ni-Sepharose 6 Fast Flow resin (Sigma-Aldrich) (resuspended in Lysis Buffer) which was then incubated at 4°C for 90 min on a vertical disc rotator. After incubation the resin was sedimented and washed four times with 1 mL Washing Buffer (4 M urea, 50 mM NaH_2_PO_4_, 300 mM NaCl, 20 mM imidazole, pH 8.0). Protein was eluted in three fractions using 0.5 mL Elution Buffer (50 mM NaH_2_PO_4_, 300 mM NaCl, 500 mM imidazole, pH 8.0).

### 2.4. Gel Electrophoresis and Western Blotting

Protein integrity and molecular mass calculation were evaluated by running samples on 12% SDS-PAGE [13]. Proteins were stained with Coomassie Brilliant Blue R-250 (Sigma-Aldrich). For Western Blotting, a monoclonal anti-poly-histidine clone His-1 alkaline phosphatase conjugate (Sigma-Aldrich) was diluted 1 : 1000 in PBS, and nitroblue tetrazolium salt and 5-bromo-4-chloro-3-indolyl phosphate (NBT/BCIP kit, Invitrogen) were used for signal development.

### 2.5. In-House Enzyme-Linked Immunosorbent Assay (ELISA)

The wells of polystyrene plates (Greiner Bio-One) were sensitized with 20 ng purified rMEHCV dissolved in 100 *µ*L 0.1 M sodium carbonate-bicarbonate buffer (pH 9.6). After incubation at 4°C for 16 h the coated wells were washed with PBST (PBS supplemented with 0.2% Tween 20, pH 7.2) and blocked for 2 h at 37°C with PBS containing 5% (w/v) dried skim milk powder and washed again with PBST. Subsequently, 100 *µ*L of a dilution (100 *µ*L PBST, 5% (w/v) dried skim milk powder, and 5 *µ*L serum) was placed into the wells resulting in a final dilution of approximately 1/20. After incubation for 1 h at 37°C, the wells were washed with PBST and 100 *µ*L of peroxidase-labeled goat anti-human immunoglobulin G conjugate (Sigma-Aldrich) diluted at 1 : 25,000 in PBS containing 5% (w/v) dried skim milk powder was added following incubation for 1 h at 37°C. The wells were again washed with 200 *µ*L OPD by incubating for 30 min at room temperature. The optical densities (OD) were read at 450 nm. The results from the in-house kit were compared to those obtained from the Hepanóstika HCV Ultra® (Beijing, China) commercial kit.

### 2.6. Circular Dichroism Spectroscopy

Circular dichroism (CD) assays were carried out using Jasco J-815 spectropolarimeter (Jasco, Tokyo, Japan) equipped with a Peltier-type temperature controller and thermostatized cuvette holder linked to a thermostatic bath. Far-UV spectra were recorded using 0.2 cm path length quartz cuvettes at a protein concentration of 0.084 mg/mL in 5 mM Tris-HCl (pH 7.0 and 8.0). Five consecutive measurements were accumulated and the averaged spectra were recorded. The observed ellipticities were converted into molar ellipticity [*θ*] based on molecular mass per residue of 115 Da [14]. The data was corrected for the baseline contribution of Tris-HCl buffer considered to estimate the secondary structure content using the CD Spectra Deconvolution (CDNN) [15]. Thermal denaturation experiments were performed by temperature increase from 25 to 95°C followed by changes in dichroic signal at 208 nm ([*θ*]_208_). The thermal denaturing curves were normalized and expressed considering the unfolded protein fraction (*f*_U_) according to (1). The equilibrium constants for unfolding process and thermodynamic parameters enthalpy (Δ*H*_*m*_), entropy (Δ*S*_*m*_), and the Gibbs free energy (Δ*G* ^25^) were calculated from (2), (3), and (4), respectively [16]:

(1) fU=yN−yyN−yU,

(2) Keq=fU1−fU,

(3) Rln⁡Keq=−ΔH1T+ΔS,

(4) ΔG=ΔH−TΔS,

where *y*_N_ and *y*_U_ represent the amount of *y* protein present in native and unfolded state, respectively. *R* is the universal gas constant (1,987 cal K^−1^ mol^−1^) and *T* the temperature in Kelvin (K). The melting temperature (*T*_*m*_), where the unfolding occurs, was calculated from the nonlinear fitting of unfolding curves using Origin software 8.0 (Microcal Software Inc., Northampton, MA program).

## 3. Results

### 3.1. Design of rMEHCV

**Figure 1:**
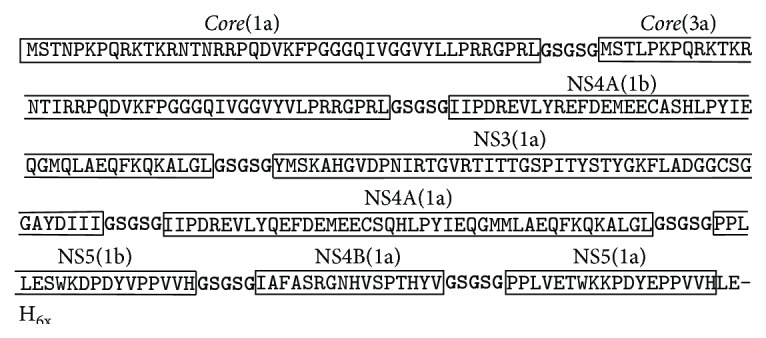
Primary structure of rMEHCV. Epitopes derived from structural (*core*) and nonstructural proteins (NS3, NS4A, NS4B, and NS5) from HCV genotypes 1, 2, and 3 are boxed. Each epitope is separated by a flexible linker (bold). A histidine tag (H_6x_) is present at the C-terminal end.

In order to design a multiepitope protein that could be of diagnostic use, linear and conserved immunodominant epitopes which are known to elicit anti-HCV antibodies were selected based on data from the literature [9–12]. These epitopes are located on five distinct regions of the HCV polyprotein. Due to the sequence variation among genotypes 1a, 1b, and 3a, eight epitopes—*core*(1a),* core*(3a), NS4A(1b), NS3(1a), NS4A(1a), NS5(1b), NS4B(1a), and NS5(1a)—were chosen representing genotypes circulating worldwide, especially in South America. Multiple sequence alignments of the HCV genotypes of different isolates allowed the identification of conserved immunodominant epitopes which were assembled* in tandem* and connected by flexible glycine-serine linkers. This would allow the epitopes to be freely available for interaction with their cognate antibodies thus contributing to the overall sensitivity and specificity of the diagnostic test. The primary amino acid sequence of rMEHCV was predicted to encode a ~34.4 kDa protein which is shown in Figure 1.

### 3.2. Expression and Purification of the rMEHCV

**Figure 2:**
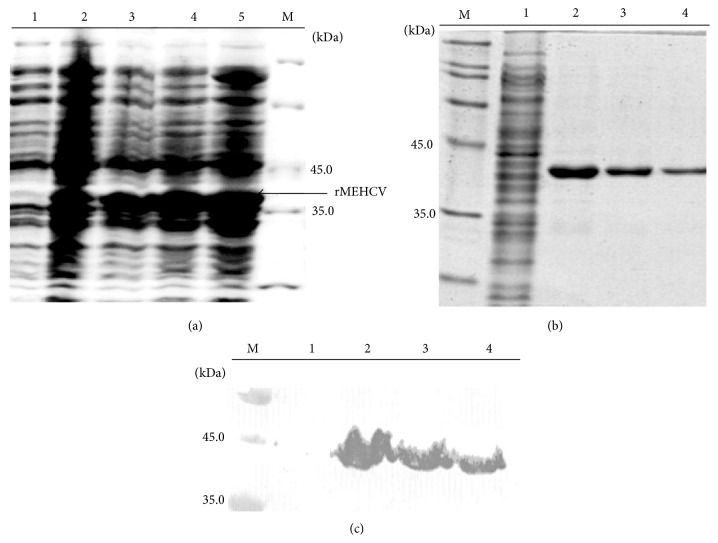
Expression and purification of rMEHCV. (a) Cell lysates from a selected clone induced by IPTG visualized on a Coomassie Blue stained 12% SDS-PAGE. Lane 1, cell lysates from uninduced culture. Lanes 2–5, cell lysates from induced culture after 2, 4, 6, and 8 h, respectively; Lane M, unstained protein molecular weight marker (Fermentas Life Sciences). The arrow indicates the position of the band corresponding to rMEHCV. (b) Fractions collected after Ni-NTA chromatography visualized on 12% SDS-PAGE. Lane M, molecular weight markers (Weight Standard, Broad Range, BioRad). Lane 1, flow-through, Lanes 2–4, purified rMEHCV after elution with 500 mM imidazole. (c) Western blot analysis of the purified rMEHCV. The monoclonal antibody, anti-polyhistidine alkaline phosphatase conjugate, was used after dilution 1 : 1000 in PBS (pH 7.2). Lane M, PAGE*™* Ruler (Fermentas Life Science). Lanes 1–4 correspond to the same fractions shown in (b).

The gene coding for rMEHCV was cloned into the bacterial expression vector pET21a for inducible expression under the control of the T7 bacteriophage promoter. After transformation of* E. coli* BL21 (DE3) a selected clone was analyzed for rMEHCV expression by SDS-PAGE after induction with IPTG. As shown in Figure 2(a), an inducible protein band with a molecular mass of ~35 kDa was observed. The cell lysate was incubated with Ni-NTA resin in the presence of 8 M urea and samples were collected during different steps of the purification and analyzed by SDS-PAGE. Elution of the bound proteins was achieved with 500 mM imidazole and resulted in highly purified rMEHCV (Figure 2(b)). The 6x histidine tag at the C-terminal end of rMEHCV was used to identify the recombinant protein by Western Blotting. The affinity-purified protein was blotted and probed with commercially available monoclonal anti-polyhistidine antibody which recognized the purified protein as being rMEHCV (Figure 2(c)).

### 3.3. Human Anti-Hepatitis C Virus Antibodies Recognize rMEHCV

**Figure 3:**
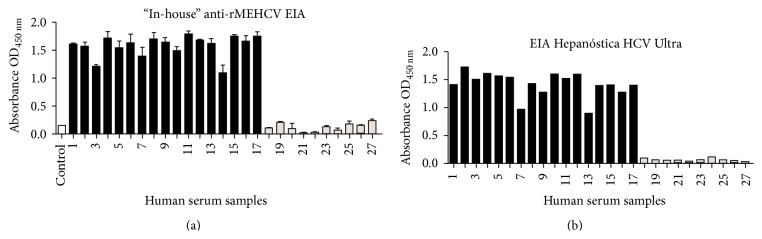
Specificity test for rMEHCV. (a) Detection of HCV using an in-house EIA. Each well of the EIA plates was coated with 20 ng purified rMEHCV and assayed with 17 positive and 10 negative human sera samples. The white bar represents the control “blank” test (0 ng protein coated + serum # 1 + conjugated secondary antibody), and the black bars and gray bars represent the sera positive and negative for anti-HCV, respectively. The bars represent the average of triplicates of each serum with its respective standard deviation. (b) Analysis of samples tested in (a) with a commercial kit (Hepanóstika HCV Ultra kit). The sera were diluted 1/10.

**Figure 4:**
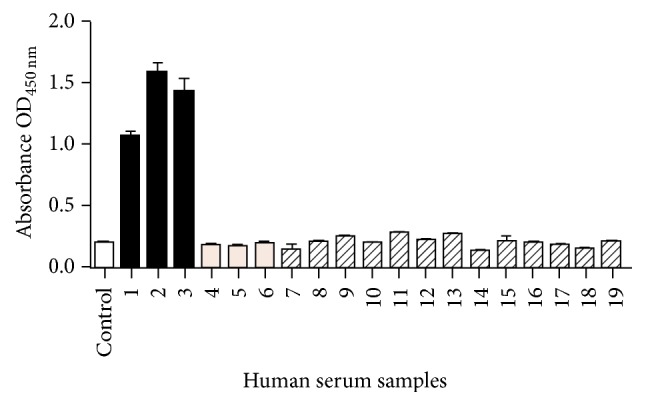
Cross-reactivity test of rMEHCV using in-house EIA. Human sera samples were diluted 1/20 and secondary antibody was diluted 1/25,000. The bars represent the standard deviation of triplicates. The white bar represents the control “blank” test (0 ng protein coated + serum # 1 + conjugated secondary antibody), and the black, gray, and crosshatched bars represent positive sera, negative for anti-HCV and positive for other diseases, respectively. Sample numbering follows the list shown in Table 1.

After protein purification, an in-house ELISA was developed for the assessment of rMEHCV as a potential antigen for HCV detection. In order to standardize the amount of protein required to obtain a suitable signal, different amounts of purified rMEHCV were coated onto ELISA plates. After blocking, 10 *μ*L sera samples (anti-HCV positive and negative) were added. The results showed that 0.02 *µ*g/mL of the recombinant protein provided the optimal signal, that is, OD > 0.8 (data not shown). Therefore, 20 ng/well (in 100 *µ*L) rMEHCV was utilized for setting up the in-house anti-HCV test kit. To establish the specificity of rMEHCV, 17 human positive and 10 negative sera samples for anti-HCV were evaluated in triplicate. The results showed that the test kit was able to distinguish positive and negative sera, showing no false-negative or false-positive results (Figure 3(a)) as compared to a commercial kit (Hepanóstika HCV Ultra) (Figure 3(b)) which essentially yielded the same results. In addition, to establish if rMEHCV does not exhibit any false-positives in the presence of sera samples from humans infected with non-HCV pathogens, 13 sera samples from patients carrying common infections, hepatitis A, hepatitis B, rubella, cytomegalovirus, and toxoplasmosis, were evaluated using the in-house anti-HCV test kit and all samples scored negative (Figure 4).

### 3.4. Structural Analysis by Circular Dichroism (CD)

**Figure 5:**
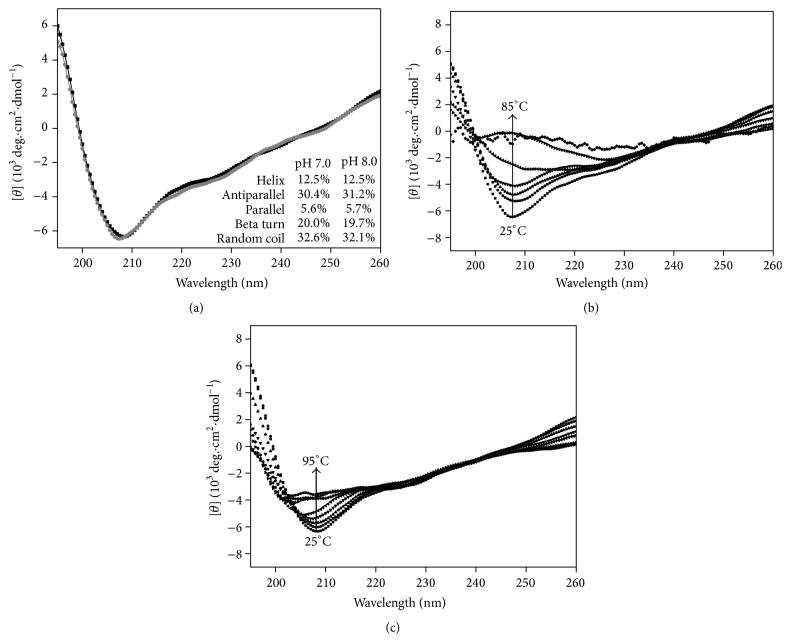
Structural analysis of rMEHCV by circular dichroism. (a) Far-UV CD spectra of rMEHCV (2.5 *μ*M) at 25°C in 5 mM Tris-HCl pH 7.0 (gray line) and pH 8.0 (black line). The table inset shows the secondary structure content of protein in both pHs; (b) Far-UV CD spectra of rMEHCV (2.5 *μ*M) in 5 mM Tris-HCl (pH 7.0) as a function of temperature. The arrow indicates the increase of temperature from 25 to 85°C, in which the complete loss of CD signal was observed; (c) Far-UV CD spectra of rMEHCV (2.5 *μ*M) in 5 mM Tris-HCl (pH 8.0). The arrow indicates the increase of temperature from 25 to 95°C, in which the CD signal reducing until ~3,500 deg·cm^2^·dmol^−1^ was observed.

**Figure 6:**
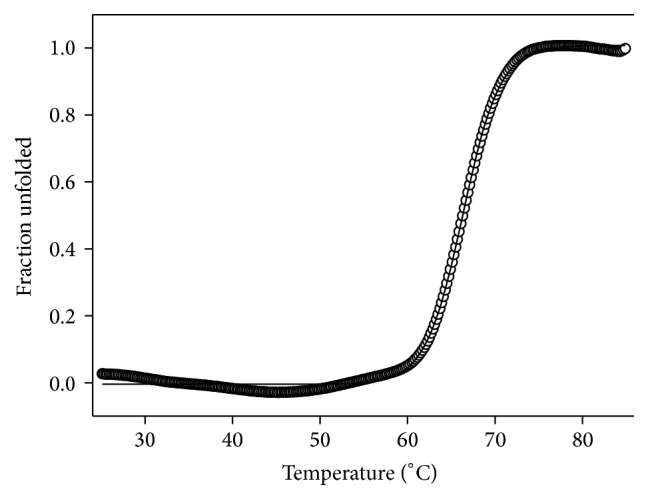
Heat-induced unfolding curve of rMEHCV (2.5 *μ*M) monitored by CD spectroscopy at 208 nm in 5 mM Tris-HCl pH 7.0. The black line corresponds to the sigmoid fitting of experimental data. The fraction unfolded data are calculated considering the changes in molar ellipticities at 208 nm and equations described in Material and Methods. The melting temperature, *T*_*m*_, corresponding to the inflection point of the sigmoid is 66.32°C.

**Figure 7:**
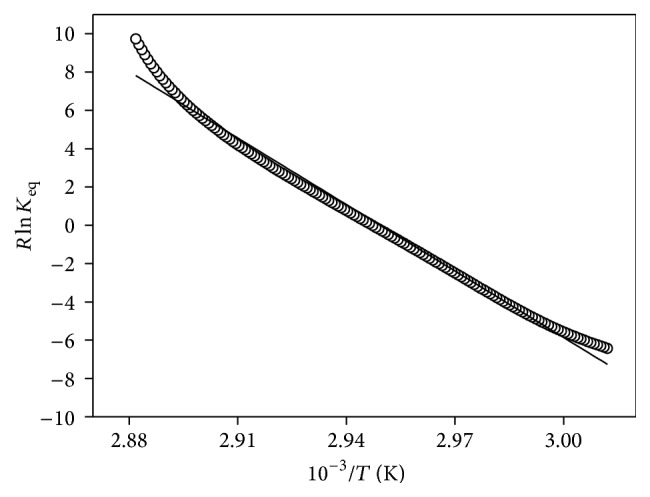
Van't Hoff plot of rMEHCV (2.5 *µ*M) in 5 mM Tris-HCl (pH 7.0). The black line corresponds to the linear fitting of the experimental data obtained from the unfolding curve.

In order to gain more insight into the structure of rMEHCV we performed CD analysis. The Far-UV CD spectra of rMEHCV at 25°C, pH 7.0 and 8.0 presented a negative dichroic band at 208 nm, a broad and of low intensity negative band at 220–228 nm, and positive prominent CD signal at 195 nm (Figure 5(a)), suggesting predominantly the presence of *β*-sheet and a low content of *α*-helix structures. It was confirmed by the estimated secondary structure contents of rMEHCV at pH 7.0 of 12.5%  *α*-helix, 56.0%  *β*-sheet (parallel, antiparallel, and turns), and 32.6% random-coil structures. At pH 8.0 almost the same pattern of secondary structure was observed, as depicted in Figure 5(a). Although the alkaline conditions did not promote considerable secondary structure alterations at 25°C, compared with those at pH 7.0, the thermal denaturation assays indicated the pH dependent structural changes of protein, as judged by differences in unfolding processes (Figures 5(b) and 5(c)). The Far-UV spectra at pH 7.0 show a gradual decrease of the dichroic signal (upward until ~zero), as a function of temperature, suggesting the whole protein unfolding process (Figure 5(b)). In contrast, despite dichroic signal decreasing from about −6,000 to −3,500 degree·cm^2^·dmol^−1^, at pH 8.0 indicating the secondary structure changes (Figure 5(c)), the whole pattern of protein denaturation could not be verified. It was in agreement with equilibrium thermal folding/unfolding process of rMEHCV from 25 to 95°C, monitored by Far-UV CD at 208 nm. At pH 7.0 the rMEHCV unfolded process occurs as two-state model from native to unfold protein (Figure 6), whereas at pH 8.0 two distinctive transitions involving the native and molten globule intermediates, but not the whole denatured protein, were observed (data not shown). The nonlinear fitted unfolding curve at pH 7.0 (Figure 6) shows the inflection points corresponding to the melting temperatures of 66.3°C. The thermodynamic parameters, calculated according to the van't Hoff approximation (Figure 7) at pH 7.0, were Δ*H*_*m*_ 115.8 kcal·mol^−1^, Δ*S*_*m*_ 341.47 cal·mol^−1^K^−1^ and the Gibbs free energy (Δ*G* ^25^) 14.02 kcal·mol^−1^ which indicates high stability of protein in this condition. Additionally, the protein seems to be more stable at pH 8.0, as indicated by the not observed unfolding pattern until 95°C under this condition.

## 4. Discussion

Hepatitis C is a worldwide public health problem. In Brazil, it has been shown that from those individuals who test positive for HCV infection approximately 80% have the chronic form of the disease. Based on these data, it is estimated that there are 400,000 to 3,800,000 cases of chronic hepatitis C in Brazil alone [8]. Because of the increase in the number of cases detected worldwide in recent years, the demand for diagnostic tests for HCV has increased accordingly. The method of choice for HCV detection is generally based on EIA because of its ease of use, low variability, easy automation, and low costs. Over the years, several generations of EIA tests have been developed with the aim of increasing sensitivity and specificity. The first generation anti-HCV tests were developed in the late 80s [17]. These tests contained a single recombinant antigen derived from the NS4 region and lacked optimal sensitivity and specificity. In order to circumvent these limitations, second generation tests contained antigens derived from the HCV* core*, NS3 and NS4 regions [18]. This resulted in higher levels of sensitivity but a small increase in specificity which nonetheless shortened seroconversion [19]. Third generation anti-HCV tests included an antigen from the NS5 region which resulted in a progressive increase in sensitivity [20] but not all patients with active infection could be identified with these tests [21].

With the advent of recombinant DNA technology, EIA tests were significantly improved because higher antigen concentration could be used. Also, due to the fact that certain antigens are not readily recognized by antiserum belonging to different serovars it is desirable that diagnostic kits should be able to detect as many genotypes as possible. Genetic information is an important parameter to direct the patients for a specific treatment. For example, treatment with interferon-*α* and ribavirin has an efficiency of 40–45% in patients infected with HCV genotype 1, whereas in those infected with genotypes 2 and 3 the efficiency increases up to 70–80% [10].

The urgent need for a diagnostic test which offers increased degrees of sensibility and specificity prompted us to develop a recombinant multiepitope protein bearing HCV-specific immunodominant epitopes. Several studies have reported the successful use of multiepitope protein for diagnosis of infectious diseases such as leishmaniasis [22], hepatitis B [23], hepatitis C [12], toxoplasmosis [24], tuberculosis [25], leprosy [26], leptospirosis [27], dengue [28], and Chagas disease [29]. A multiepitope protein (r-HCV-F-MEP) for hepatitis C diagnosis has been previously developed bearing 5 immunodominant regions comprising genotypes circulating worldwide and one Indian isolate [12]. From a clinical perspective, the multiepitope protein developed in our work (rMEHCV) aimed at the detection of the most representative HCV serotypes particularly found in Brazil. This was achieved by the inclusion of sequences from the* core*, NS3, NS4A, NS4B, and NS5 regions from genotypes 1a, 1b, and 3a. Genotypes 1, 2, and 3 are found in all continents and constitute the majority of HCV isolates [9]. Genotype 4 is more common in the North and Center-West Africa, while genotypes 5 and 6 are most common in South Africa and Asia, respectively [10]. In Brazil, genotypes 1 and 3 are the most prevalent [11].

We based our construct on the immunodominant regions previously proposed [12] but focused on genotypes 1a, 1b, and 3a. Furthermore, we include extra copies of the immunodominant regions from proteins NS4a and NS5 in order to cover both genotypes 1a and 1b which have some sequence differences in these particular regions.

Since the major goal of this study was to develop a recombinant protein for use in diagnostic kit, the ability of rMEHCV to detect anti-HCV antibodies was tested in an in-house EIA. In this assay the recombinant protein was used as the capture antigen and human sera samples infected or not with HCV were tested. Our results showed that rMEHCV was recognized by all HCV-infected samples with a 100% agreement with a commercial kit. In addition, when exposed to sera samples from patients having other (non-HCV) infections no cross-reaction was observed, thus demonstrating the specificity of rMEHCV, a desirable feature for HCV diagnosis.

The secondary structure content and structural stability of rMEHCV under different pH and temperatures were also studied. These parameters are important given that epitopes should be stable under diagnostic assay conditions. The structural stability of rMEHCV was investigated by circular dichroism spectroscopy in neutral and alkaline conditions. It is known that the CD spectrum of the typical *α*-helix exhibits two prominent negative bands. One of them occurs at 208 nm, generally of reduced intensity in short helices, and the other at 222 nm, related to strong hydrogen-bonding environment and independent of the length of the helix. The typical *β*-sheet proteins exhibit a negative band near 218 nm and a positive band near 195 nm, in which the position and magnitude are generally variable. In contrast, unordered polypeptides exhibit a negative band near 200 nm [14, 30]. In this work, the Far-UV CD spectroscopy results indicate that rMEHCV is a structured protein at neutral and alkaline conditions. It seems to contain a small amount of helical structure with low intensity CD signal at 222 nm and high amount of *β*-sheet structures, indicated just by the positive band at 195 nm, once it does not present the typical maximum at around 218 nm (Figure 5(a)).

While rMEHCV exhibits similar amount of secondary structure at pH 7.0 and 8.0 (Figure 5(a)), the thermal-induced conformational transitions were much less for the latter indicating more stability at pH 8.0 (Figure 5(c)) than at pH 7.0 (Figure 5(b)). The CD_208nm_ measurement of rMEHCV at pH 7.0 (Figure 6) revealed a typical thermal reversible two-state transition from native to unfolded state [16, 30, 31]. It was also indicated by CD rescanning under protein sample cooling (95 to 25°C), after its complete thermal unfolding until 95°C (data not shown). The high values of thermodynamic parameters obtained from the unfolded curve at this pH 7.0, mainly Δ*G* ^25^ of 14.02 kcal·mol^−1^, indicate a remarkable stability of rMEHCV. It depends on the enthalpy changes that correspond to the binding energy of noncovalent interactions, and the entropy changes associated with the increase of conformational freedom in the polypeptide chain and hydration of exposed groups on unfolded state. Furthermore, the transition temperature (*T*_*m*_) from native to unfolded state occurs at temperatures above 66°C, compatible with the high stable thermophilic proteins [32, 33]. As seen in this neutral condition the protein was completely unfolded at 95°C. In contrast, at pH 8.0 the conformational changes of the protein could be verified throughout the temperature range of 25 to 95°C (Figure 5(c)), which preserve part of its secondary structure despite temperature increase. This unfolding process involves the presence of intermediates that is larger than the native protein and has an intact secondary structure, known as a molten globule state [31, 34], indicated through the maintenance of dichroic signal of −3,500 degree·cm^2^·dmol^−1^ at 95°C. It is known that the presence of molten globule intermediate in unfolded process depends not only on the amino acid composition and protein structural arrangement but also on the environmental conditions [30, 31, 34]. Overall, the most abundant amino acids residues composing rMEHCV are glycine (14.6%) and proline (9.2%) which could in part explain the high stability of the protein at both pHs due to favoring of high content of polypeptide fold in globular protein. Furthermore, protein stability can be also explained by two main points: (i) the structural arrangement of rMEHCV due to differences in charged residues as a function of pH; (ii) the high stability at pH 8.0, which is the closest pH to the theoretical isoelectric point of the protein (pI of ~9.0—http://web.expasy.org/protparam/), where globular proteins tend to present maximum stability [16, 35, 36]. The results presented here indicated that the net charges and ionic pairs, due to the high content of charged amino acid residues, induced on the pH 8.0, could also favor a more compact state. This condition results in the stabilization of the protein as a molten globule state, even at the high temperature of 95°C.

It is noteworthy that the N-terminus of rMEHCV contains 28/37 of the total number of lysine and arginine residues in the protein, while the C terminus has 11/14 histidine residues and 18/30 of the acidic residues. At neutral pH, most of these residues are charged, whereas at pH 8.0 all histidines are uncharged. The highest conformational stability of rMEHCV near the pI is likely the result of protein self-association tendency driven by favorable electrostatic interactions on the molecule surface. Additionally, the difference of stability at pH 7.0, compared to pH 8.0, may be also due to the charge balance resulting from histidine residues ionization in the unfolded state relative to the native state, and the possible high number of ionic pairs.

Therefore, we have shown that the secondary structure of rMEHCV in both pHs at 25°C was similar; however the protein was more stable at pH 8.0 as compared to neutral pH. The molecule unfolded at 95°C and at neutral pH, but it can assume an intermediate molten globule structure and a compact denatured state with significant secondary or tertiary structure at pH 8.0. Together, the results presented here showed that rMEHCV is a highly thermal stable protein at neutral and alkaline conditions and could be used under those conditions for HCV diagnosis.

## 5. Conclusions

The high epitope density derived from different HCV genotypes coupled with a simple purification procedure prompts rMEHCV as a promising alternative for hepatitis C diagnosis, with potential for development of an inexpensive diagnostic test with high degree of specificity.

## Figures and Tables

**Table 1:** Infected human sera used in this work.

Sera	Pathologies
1, 2, and 3	HCV (+)
4, 5, and 6	HCV (−)
7 and 8	HAV (hepatitis A)
9, 10, and 11	HAV and HBV (hepatitis B)
12	HBV
13 and 14	RUB (rubella) and CMV (cytomegalovirus)
15	HBV
16	RUB, CMV, and TOX (toxoplasmosis)
17	RUB and TOX
18	CMV and TOX
19	HBV
